# Impact of intervention on healthcare waste management practices in a tertiary care governmental hospital of Nepal

**DOI:** 10.1186/1471-2458-14-1005

**Published:** 2014-09-26

**Authors:** Binaya Sapkota, Gopal Kumar Gupta, Dhiraj Mainali

**Affiliations:** Government of Nepal Civil Service Hospital, Minbhawan, Kathmandu, Nepal; Janta National Hospital, Damak, Jhapa, Nepal

**Keywords:** Committee, Healthcare waste management, Policy, Standard operating procedure, Training

## Abstract

**Background:**

Healthcare waste is produced from various therapeutic procedures performed in hospitals, such as chemotherapy, dialysis, surgery, delivery, resection of gangrenous organs, autopsy, biopsy, injections, etc. These result in the production of non-hazardous waste (75–95%) and hazardous waste (10–25%), such as sharps, infectious, chemical, pharmaceutical, radioactive waste, and pressurized containers (e.g., inhaler cans). Improper healthcare waste management may lead to the transmission of hepatitis B, Staphylococcus aureus and Pseudomonas aeruginosa.

**Methods:**

This evaluation of waste management practices was carried out at gynaecology, obstetrics, paediatrics, medicine and orthopaedics wards at Government of Nepal Civil Service Hospital, Kathmandu from February 12 to October 15, 2013, with the permission from healthcare waste management committee at the hospital. The Individualized Rapid Assessment tool (IRAT), developed by the United Nations Development Program Global Environment Facility project, was used to collect pre-interventional and post-interventional performance scores concerning waste management. The healthcare waste management committee was formed of representing various departments. The study included responses from focal nurses and physicians from the gynaecology, obstetrics, paediatrics, medicine and orthopaedics wards, and waste handlers during the study period. Data included average scores from 40 responders. Scores were based on compliance with the IRAT.

**Results:**

The waste management policy and standard operating procedure were developed after interventions, and they were consistent with the national and international laws and regulations. The committee developed a plan for recycling or waste minimization. Health professionals, such as doctors, nurses and waste handlers, were trained on waste management practices. The programs included segregation, collection, handling, transportation, treatment and disposal of waste, as well as occupational health and safety issues. The committee developed a plan for treatment and disposal of chemical and pharmaceutical waste. Pretest and posttest evaluation scores were 26% and 86% respectively.

**Conclusions:**

During the pre-intervention period, the hospital had no HCWM Committee, policy, standard operating procedure or proper color coding system for waste segregation, collection, transportation and storage and the specific well-trained waste handlers. Doctors, nurses and waste handlers were trained on HCWM practices, after interventions. Significant improvements were observed between the pre- and post-intervention periods.

## Background

Healthcare waste is produced from various therapeutic procedures in hospitals, such as chemotherapy, dialysis, surgery, delivery, resection of gangrenous organs, autopsy, biopsy, injections, etc. These result in the production of non-hazardous waste (75–95%) and hazardous waste (10–25%), such as sharps, infectious, chemical, pharmaceutical, radioactive waste, pressurized containers (e.g., inhaler cans) [1–12]. Non-hazardous wastes, from healthcare settings, are comparable to household waste, in regards to their risk to both human health and the environment. Similar disposal processes can be applied to both household waste and non-hazardous healthcare waste [3, 8, 13, 14]. If non-hazardous waste is mixed with hazardous waste, disposal should be employed as per regulations for hazardous waste [3]. Improved segregation of waste would thus minimize the burden of total hazardous waste [14].

Improper healthcare waste management causes environmental pollution, and infectious waste may lead to the transmission of more than 30 significant pathogens, including typhoid, hepatitis B, hepatitis C, HIV, Escherichia coli, Staphylococcus aureus, and Pseudomonas aeruginosa [1, 2, 4, 5, 11–21]. When healthcare waste is placed in landfills or buried, contamination of ground water may occur, which may result in the spread of E. coli. Pathogens, present in waste, can also enter and remain in the air for a long period, in the form of spores or pathogens. Hence, healthcare establishments should enhance the practice of waste segregation, sorting and resource recycling and recovery [22]. Furthermore, proper waste treatment methods not only decrease the weight, and volume of the waste but also the infectivity and organic compounds in the waste [9].

Poor healthcare waste management (HCWM) practices may result in patients, staff, waste handlers and the community being exposed to the unnecessary health risks of the waste [19]. In developing countries, HCWM has not gained much momentum, and healthcare waste is frequently disposed along with domestic waste [5, 13, 23]. Improper HCWM practice is alarming in developing countries because resources are inadequate to manage wastes, and waste management is often delegated to poorly educated and untrained laborers, who perform without proper guidance or adequate protection [11, 16].

Many studies have focused on healthcare waste management practices in Jordan, Iran, Egypt, Saudi Arabia, Kuwait, Tanzania, Mauritius, Netherlands, Finland, Korea, Turkey, Brazil, Mongolia, Greece, USA, UK, China, Bangladesh and India. In Bangladesh, Nigeria, Iran, Jordan, Libya, Botswana and India, there is a lack of legal provisions, rules and regulations regarding healthcare waste management, suitable waste treatment facilities, protective measures and efficient training [1, 5, 7, 12, 18, 20, 24]. Our study is the first study in Nepal to evaluate the impact of pre- and post-test HCWM interventions and will help policy makers devise effective waste management regulations to protect both the people and environment of Nepal.

## Methods

### Study site and duration

The study was conducted at Government of Nepal Civil Service Hospital from February 12 to October 15, 2013. It is a 132-bed tertiary care general hospital, situated at Minbhawan, Kathmandu. The annual outpatient flow at the hospital is 255,000 and inpatient flow is 35,000.

### Study design

This was a pre- and post-test interventional study to determine the impact of healthcare waste management (HCWM) practices, including its collection, segregation, transportation, treatment and ultimate disposal procedure.

### Ethical consideration

Civil Service Hospital Health Care Waste Management Committee granted permission for the study to be conducted. The ethical nature of the study was approved by Norvic International Hospital Ethical Review Committee.

### Study procedure

Regular visits to gynaecology, obstetrics, paediatrics, medicine and orthopaedics wards were performed twice daily by the researchers to monitor how HCWM was practiced. The first visit was from 9:00 to 10:00 in the morning and the second visit from 16:00 to 17:00 in the afternoon. These wards were chosen as model wards to explore the feasibility and sustainability of waste management program throughout hospital in the later stage.

Data were collected on a daily basis regarding where waste generation, collection, segregation, storage, transportation, and treatment, both on- and off-site. Information was also collected from the nurses, physicians and waste handlers in the gynaecology, obstetrics, paediatrics, medicine and orthopaedics wards. Data included average scores from 40 responders. Scores were based on the compliance with the IRAT (Table 1).

**Table 1:** **Pre-interventional evaluation of Healthcare waste management practices**

SN	Questions		Pre-intervention		Post-intervention	
		Weight value for pre- and post-intervention	“Y” or “N”	Score	“Y” or “N”	Score
	**Part I. Initial interview**					
	**Organization**					
1	In-charge of HCWM	5	N	0	Y	5
2	Permanent committee that deals with HCWM and meets on a regular basis	1.5	N	0	Y	1.5
3	Roles and responsibilities regarding HCWM made clear to the staff	1.5	N	0	Y	1.5
	**Policy and Planning**					
4a	HCF has written policies dealing with HCWM.	2	N	0	Y	2
4b	HCF has written plans, manuals, or written procedures dealing HCWM.	2	N	0	Y	2
5	Policies, plans, manuals, and/or written procedures consistent with national laws, regulations, and any permits.	3.5	N	0	Y	3.5
6	HCF has a plan for recycling or waste minimization.	1.5	N	0	Y	1.5
7	HCF policy explicitly mentions a commitment to protect the environment.	0.5	N	0	Y	0.5
8	HCF is mercury-free. OR HCF has a policy or plan to phase out mercury.	1.5	N	0	Y	1.5
	**Training**					
9	HCF has a training program on HCWM for managers, health professionals, waste workers, and auxiliary staff.	5	N	0	Y	5
10	Training program includes relevant national laws and regulations.	1	N	0	Y	1
11	Training program includes segregation, collection and handling of sharps waste, use of proper containers and bags for infectious waste, color coding, 3/4th fill rule, use of personal protection equipment by waste workers, transport, storage, and treatment	2	N	0	Y	2
12	Staffs are trained, including new staff when they begin their employment.	3	N	0	Y	3
13	Refresher training at least once a year	1	N	0	Y	1
	**Occupational Health and Safety**					
14	Policies and plans related to HCWM include occupational health and safety (including policies for NSI or exposure to blood splatter). OR HCF has separate occupational health and safety policies that include needle-sticks and exposure to blood.	3	N	0	Y	3
15	Workers who collect, transport and treat waste are provided with proper PPE (gloves, shoes or boots, and aprons)	2	N	0	Y	2
16	Health workers and workers handling waste are given hepatitis and tetanus vaccinations.	2	N	0	Y	2
	**Monitoring, Evaluation and Corrective Action**					
17	System of internal monitoring or inspection to determine compliance with HCWM requirements.	1	N	0	Y	1
18	System of taking corrective action when practices or technologies related to HCWM do not meet the requirements.	1	N	0	Y	1
19	Policies and/or plans are reviewed or updated at least once a year.	0.5	N	0	Y	0.5
	**Financing**					
20	HCF has an annual allocation in its budget for HCWM.	4	Y	4	Y	4
21	Current budget is sufficient for HCWM.	2	N	0	Y	2
22	HCF has a long-term financing plan or mechanism to cover the costs for sustainable HCWM.	0.5	N	0	Y	0.5
	**Part II. Post-Inspection Tour Interview**					
	**Classification and Segregation**					
23	Wastes are properly segregated at the source according to different categories.	5	N	0	Y	5
24	Health workers are familiar with the classification and segregation requirements.	2	N	0	Y	2
	**Waste Generation Data**					
25	Amounts of total waste and infectious waste produced per day has been measured.	1	N	0	N	0
	Percentage of infectious waste relative to total waste	0.5	N	0	N	0
	Kilograms unrecycled waste per bed per day	0.5	N	0	N	0
	**Collection and Handling**					
26	Used syringe needles are collected without recapping.	2	N	0	Y	2
27	Sharps waste are collected in sharps containers or destroyed using needle destroyers.	5	N	0	Y	5
28	Sharps containers are puncture-resistant and leak-proof. OR Needle destroyers are approved under existing regulations or standards.	2	N	0	Y	2
29	Sharps containers are filled only 3/4th full. OR Needle destroyers are well maintained.	2.5	N	0	Y	2.5
30	Sharps containers or needle-destroyers are always available.	1	N	0	Y	1
31	Sharps containers or needle-destroyers are properly placed such that they are easily accessible to personnel and located as close as possible to the immediate area where the sharps are used.	1.5	N	0	Y	1.5
32	Health workers know what to do in the event of a needle-stick injury. OR Health workers are familiar with the policy on NSI.	1	N	0	Y	1
33	Plastic bags are used for non-sharps infectious waste of good quality. OR Specialized containers that are disinfected, cleaned and reused and do not require plastic bags are used.	1	Y	1	Y	1
34	Plastic bags are always available. OR Specialized containers described in #33 are always available.	1	Y	1	Y	1
35	Bag holders or hard containers holding the plastic bags are of good quality. Specialized containers that are disinfected, cleaned and reused and do not require plastic bags are used.	0.5	Y	0.5	Y	0.5
36	Infectious wastes are removed at least once a day.	1	Y	1	Y	1
37	Waste workers know what to do if sharps or infectious waste is accidentally spilled. OR Waste workers are familiar with the spill clean-up plans.	0.5	N	0	Y	0.5
	**Color Coding and Labeling**					
38	HCF uses a system of color coding for different types of wastes.	3	N	0	Y	3
39	Colors of the waste containers are consistent with the color coding.	2	N	0	Y	2
40	Infectious waste bags are colored or labeled in accordance with the policies or regulations	1	N	0	N	0
	**Posters or Signage**					
41	posters or signs showing proper segregation of healthcare waste	0.5	N	0	Y	0.5
	**Transportation Inside Health Establishment**					
42	Waste is transported away from patient areas and other clean areas.	0.5	Y	0.5	Y	0.5
43	Waste is transported in a closed (covered), wheeled transport cart.	1	N	0	Y	1
44	Transport cart is cleaned at least once a day.	0.5	N	0	Y	0.5
	**Storage**					
45	Storage area meets the proper requirements.	1	N	0	Y	1
46	Storage area is kept clean.	0.5	N	0	Y	0.5
47	Wastes are removed before the maximum allowable storage time is exceeded.	1	N	0	Y	1
	**Hazardous Chemical, Pharmaceutical and Radioactive Waste**					
48	Hazardous chemical, pharmaceutical, and radioactive wastes are segregated from infectious and general non-risk wastes.	4	N	0	Y	4
49	HCF has a plan for treatment and disposal of hazardous chemical, pharmaceutical, and radioactive wastes.	1	N	0	Y	1
	**Treatment and Disposal**					
50	HCF treats its infectious waste (either on-site or at an off-site treatment facility) before final disposal.	25	Y	25	Y	25
51	Laboratory cultures and stocks of infectious agents are treated within HCF before being taken away from the facility.	2	Y	2	Y	2
52	Contingency plan for treatment of infectious waste in the event that the treatment technology is shut down for repair.	1	N	0	Y	1
53	Waste is transported safely to the treatment area.	0.5	Y	0.5	Y	0.5
54	Treatment area is located in a place that is easily accessible to the waste worker but not accessible to the general public.	0.5	Y	0.5	Y	0.5
55	HCF has a program of regular inspection and periodic maintenance of the treatment technology.	3	N	0	Y	3
56	Treatment system is clean, operating properly, and well maintained.	3	N	0	Y	3
57	Treatment system destroys or mutilates sharps waste in order to prevent reuse.	1	N	0	N	0
58	HCF uses an approved non-incineration treatment technology such as an autoclave-shredder, integrated steam treatment system, or microwave unit.	6	N	0	N	0
59	Incinerator meets international standards.	3	N	0	N	0
60	PVC plastics are kept out of the waste that is burned.	1	N	0	N	0
61	Waste that is treated in an alternative technology is disposed of in a sanitary landfill. OR Incinerator ash is buried in a hazardous waste landfill.	1	N	0	N	0
	**Wastewater**					
62	HCF treats its wastewater (liquid waste) before being released. OR HCF is connected to a sanitary sewer that is linked to a wastewater treatment plant.	3	N	0	N	0
63	Treated wastewater from HCF meets national or international standards.	1	N	0	N	0
	**Total Score**	142		36		123

### Interventions provided

The healthcare waste management committee (HCWMC) was formed under the leadership of the executive director of the hospital. The director was responsible for the overall activities of the committee, including managing annual budget allocation for HCWM activities, and equipment purchasing from internal sources of the hospital. The members of the committee were representatives from all departments and units of the hospital, which were responsible for waste generation. The written HCWM policy and standard operating policy (SOP) were developed and endorsed by the committee. They were consistent with national laws and regulations, such as Solid Waste Management Act 2011; Environmental Protection Act 1997; Environmental Protection Rules 1997; and Solid Waste Management Policy 1996. The committee forwarded them to the director and he finally approved them. The HCWM policy was one aspect of the hospital infection prevention and control policy. The hospital also established HCWM Unit to monitor all HCWM activities in the hospital. The hospital had been following the government rules and regulations, but had not developed its own policy and SOP prior to this.

Roles and responsibilities of the committee members were assigned in the policy. The member-secretary of the committee was selected as the focal person, for reporting and disseminating HCWM related issues and arranging meeting of the committee every month. The committee also assigned a waste management officer for day-to-day internal monitoring of waste management practices.

Training program, related to HCWM, was conducted for physicians, nurses and waste handlers. The program was based on safe HCWM practices, recommended by World Health Organization (WHO). The program included orientation to HCW and its management, standard operating procedure and legal provisions for the safe waste management, segregation, collection and handling techniques of various waste, as well as occupational health and safety issues, safe injection practices [9].

The committee appointed a focal nurse from respective wards, each month. Focal nurse would be responsible for counseling and recording HCWM related activities. She would be selected by the HCWM meeting, on rotation basis. She would submit the record weekly to her ward in-charge, and then finally to the HCWM Committee. She was given HCWM related written job description of counseling caregivers of patient about the objectives, procedures, systems and benefits of proper HCWM, with demonstration. The HCWM brochure was developed by the committee, and was provided by the focal nurse to the caregivers at the time of admission of the patient. The brochure contained complete information about waste management protocol of the hospital.

The open area container, kept close to the patients’ area, contained general or non-hazardous waste. The closed area container, kept isolated from the patients’ access, contained infectious and hazardous waste. General buckets, used prior to the intervention, were replaced by the WHO- recommended polypropylene coloured buckets. Three such buckets were kept in the patients’ area (green for biodegradable waste, blue for plastic waste and black for paper waste), and two such buckets were isolated from patients’ access (red for incinerable waste and yellow for autoclavable waste). Pictorial text was placed above the bucket. Patient awareness notice board was kept above that picture, for the convenience of patients’ caregivers.

The waste collected was transported to the designated storage area, by two separate and dedicated trolleys- one for the risk waste and other for the non-risk waste. This was performed on each shift by the well-trained waste handlers, who were trained in handling, transporting, transportation schedule and route, importance of wearing personal protective equipment. They were vaccinated against hepatitis B and tetanus. They were also serologically screened for seroconversion. The first shift, of onsite waste transportation, was from 7 to 8 AM, and the second shift was from 6 to 7 PM. The contents of the bins were poured out for autoclaving or incineration. The bin was cleaned and disinfected with 0.5% sodium hypochlorite solution, once in 24 hours, as the post-treatment rinse. Sharps collected were disposed daily on sharp pit. All wastes, placed in yellow containers, were autoclaved at 121°C temperature and 15 psi pressure for 30 minutes. The waste, collected at red bucket, was incinerated, twice weekly. Incinerator, at the hospital, was double-chambered, operated at temperature between 900°C and 1200°C. The height of the chimney of the incinerator was 20 meter. The ash produced after incineration was land filled.

### Study tool and data analysis

The Individualized Rapid Assessment tool (IRAT), developed by the United Nations Development Program Global Environment Facility project, was used as the tool to collect information about HCWM practices before study and after eight months of the study. The IRAT tool resulted in an overall score, and it was designed for use by the technical consultants and/or hospital personnel, specializing in healthcare waste management. The average score of 40 respondents was processed, for final data analysis. The observation, during the assessment visit, and questionnaire filled after the assessment by the researchers, were subjected in the IRAT form. Then the IRAT automatically computed a final score. The higher the final score, the better was the HCWM system of the hospital.

The score of the IRAT analysis gave an insight on the status of HCWM at the hospital (Table 1). The score was converted into percentage. The “Yes or Y” represented the facility available and “No or N” represented the facility not available. Based on percentage, sites were further categorized as 0-25% (very poor), 26-50% (poor), 51-75% (good), and 76-100% (excellent). The pre-intervention and post-intervention scores were verified by the HCWM committee, as a means of quality assurance of the intervention, and to determine the sustainability of the improvements in the future.

## Results

The pre-intervention evaluation showed that the hospital had not allocated budget for proper waste management practices. The waste was transported away from the patients’ areas, and other clean areas. The hospital had incinerator in a location accessible to the waste handlers, but not to the public. The hospital did not have HCWM Committee, policy, standard operating procedure (SOP) and proper color coding system for waste segregation, collection, transportation and storage, as well as the specific well-trained waste handlers.

The post-intervention evaluation showed that waste water was still not treated by the hospital. The HCWM policy and SOP were developed, after interventions, and they were consistent with the national and international laws and regulations. The committee developed a plan for recycling or waste minimization. Health professionals, such as doctors, nurses and waste handlers, were provided training on HCWM practices. The training programs included segregation, collection and handling, transportation, treatment and disposal of the waste, as well as occupational health and safety issues. The committee developed a plan for treatment and disposal of chemical and pharmaceutical waste.

The pre-intervention evaluation showed that outcome of the study was poor (score 26%). The post-intervention evaluation revealed that outcome of the intervention was excellent (score 86%).

## Discussion

The pre-intervention evaluation showed the poor status of waste management practices. This might be due to the lack of environment-friendly technology and the principle of ‘reduce, reuse and recycle policy’. The committee adopted the practice of collecting and storing all healthcare wastes in a temporary waste storage area, within the premises, until they were transported to the waste treatment area [1, 5]. The waste treatment area was isolated from the patients’ treatment area, and was not accessible to the public. The treatment area consisted of waste segregation area, separate incinerator area, cytotoxic waste collection area, autoclave area, and mercury collection house. The committee organized training programs for nurses, and committee members regarding the impact of mercury and importance of its safe disposal. The used needles were cut by the needle cutter, attached with the procedure trolley. The cut portions of needle, and other sharp wastes, were kept in a bucket, half filled with 0.5% sodium hypochlorite, for 24 hours. Then they were collected in sharp pit, near waste treatment area. The committee also developed instructive posters regarding proper waste management technique to make the public aware of the effective segregation of waste. These were inexpensive, but impressive methods of gaining public support for waste management activities, and could be achieved within short period, with the limited resources available [15].

The committee adopted the system of segregation of waste at source, into suitable colour-coded high density polyethylene bags and bins, for the easy identification and segregation of infectious and non-infectious wastes. Infectious waste was packaged to protect the waste handlers and the public from the potential injury and the spread of disease [5]. Waste minimization and disposal system, including the principle of ‘reduce, reuse and recycle’, was developed, by the committee, to maintain clean environment in the hospital premises. The committee selected one focal nurse from each ward, every month, on rotation basis. The nurse was given HCWM related specific job descriptions of counseling each caregiver of patient regarding proper waste management technique. The nurse was suggested to document all counseling sessions, and provide patient information leaflet, developed by the committee. The nurse also presented the progress report and any problems, in each meeting of the committee. The committee revised the waste management policy and SOP once, and organized refresher training for all health professionals till date, and this would be continued every year.

Full set of personal protective equipment (PPE), with gloves, mask, shoes and apron, was provided to the waste handlers because lack of sufficient PPE, and knowledge regarding correct usage and benefits of using PPE, might expose them to infections and injury [2]. The waste handlers, nurses and all healthcare workers were immunized against hepatitis B thrice (initially, on first month, and after six months), and then 2–3 months later, screening for seroconversion. The booster dose would be given after 5 years. Tetanus vaccination was given twice (initially, and on first month), keeping in consideration of allergic responses to it. Hospital documented all the immunization records and distributed the immunization cards.

The committee adopted the policy of incinerating hazardous waste for the interim period, until they would be subjected to the alternative waste management techniques, such as microwaving with shredding, full range of autoclaving or chemical sterilization. If the incinerator was operated properly (high and continuous temperature, filtration of particulate emission), it would not incur excessive risks [5]. However, after incineration, combustible components of the wastes would be converted into the gaseous byproducts (carbon dioxide, carbon monooxide, dioxin, furan), and the non-combustible components would remain as solid byproduct, namely ash [16]. Moreover, the incinerator ash would be hazardous due to the presence of needles and sharps, non-destroyed pathogens, and the hazardous substances. There would also be the risk of direct inhalation by the hospital staff and patients up to a distance of 20–50 m of incinerator. Canada, USA, and Greece ceased the use of the hospital waste incinerators due to the risk of air pollution, and adopted the alternative waste disposal techniques, such as autoclaving and microwave sterilization [11].

In the long-term, the committee adopted the policy of partial cost recovery from the proper waste management. At the end of eight months of waste management practices, the committee gained partial success towards this. The committee expanded waste management practices in the whole hospital within 13.5 months of the commencement of HCWM program. The committee would start measuring the infectious and the total waste produced in kilogram per procedure per day, for their better quantification.

After the implementation of HCWM program, changes were documented via same IRAT form, which was used during pre-intervention period. The encouraging result of the post-intervention evaluation might be due to the regular monitoring and evaluation, because good HCWM depended on the dedicated waste management team, good administration, careful planning, sound organization, adequate budget allocation and the enthusiastic participation by the trained staff [17, 25]. All these shifted the poor outcome of the pre-intervention period (26% score) to the excellent outcome during post-intervention period (86% score) (Table 2).

**Table 2:** **Pre-post comparative evaluation of healthcare waste management practices**

SN	Timing of study	Maximum score	Score	Percentage score	Outcome of study
1	Pre-interventional evaluation	142	36	26%	Poor
2	Post-interventional evaluation	142	123	86%	Excellent

The results of the research emphasized that wastes should have been properly segregated at source, according to different categories, to minimize the burden of the infectious and the hazardous waste. The workers, who collected, transported and treated waste, should have been provided with the proper personal protection equipment (gloves, shoes or boots, and aprons). The health workers and the waste handlers should have been given hepatitis and tetanus vaccinations.

### Study limitation

The study was limited to a single hospital only. The only one (post eight months) survey was performed. The sample size might not be the exact representatives of the whole case so as to generalize the findings of the study.

## Conclusions

During pre-intervention period, hospital did not have HCWM Committee, policy, standard operating procedure, and proper color coding system, for waste segregation, collection, transportation and storage, as well as the specific well-trained waste handlers. The HCWM policy and SOP were developed, after interventions, and they were consistent with the national and international laws and regulations. Health professionals, such as doctors, nurses and waste handlers, were trained on HCWM practices. The pretest and posttest evaluation of healthcare waste management practices, at the hospital, showed that poor outcome of the pre-intervention study (26% score) was converted to the excellent outcome during post-intervention period (86% score).
